# Dynamic changes in sleep architecture in a mouse model of acute kidney injury transitioning to chronic kidney disease

**DOI:** 10.3389/fnins.2025.1581494

**Published:** 2025-07-03

**Authors:** Naoko Hayashi, Yuto Okabe, Kaeko Tanaka, Mina Kitajima, Keisuke Taniguchi, Motoko Yanagita, Yu Hayashi

**Affiliations:** ^1^Department of Biological Sciences, Graduate School of Science, The University of Tokyo, Tokyo, Japan; ^2^Department of Human Health Sciences, Graduate School of Medicine, Kyoto University, Kyoto, Japan; ^3^Department of Nephrology, Graduate School of Medicine, Kyoto University, Kyoto, Japan; ^4^Institute for the Advanced Study of Human Biology (WPI-ASHBi), Kyoto University, Kyoto, Japan; ^5^International Institute for Integrative Sleep Medicine (WPI-IIIS), Tsukuba Institute for Advanced Research (TIAR), University of Tsukuba, Ibaraki, Japan

**Keywords:** sleep, acute kidney injury, chronic kidney disease, mouse model, EEG

## Abstract

**Introduction:**

Sleep disorders are common in individuals with kidney failure. Whether kidney impairment is the direct cause of sleep abnormalities is unclear, however, partly due to confounding factors including comorbidities, dialysis, and drugs.

**Methods:**

Here, we used a mouse model of acute kidney injury (AKI) transitioning to chronic kidney disease (CKD) induced by aristolochic acid to examine the effects of kidney impairment on sleep architecture. Each group, comprising 8~10 male mice, underwent cortical electroencephalogram (EEG) and electroencephalogram (EMG) recordings to measure sleep and cortical oscillations.

**Results:**

During the acute phase, which models AKI, mice exhibited an approximately 20% increase in non-rapid eye movement sleep (NREMS) amount but reduced NREMS delta power in the EEG, which might be a consequence of systemic inflammation. Notably, in the chronic phase, which models CDK, the NREMS abnormalities were resolved, but rapid eye movement sleep (REMS) amount was largely reduced by approximately 20%. In addition, EEG theta power during both wakefulness and REMS was decreased. EEG slowing during wake and REMS was observed during both AKI and CKD. REMS disturbances in CKD mice correlated with serum levels of creatinine, urea nitrogen, and calcium.

**Discussion:**

Together, these findings provide direct evidence that kidney impairment has dynamic effects on sleep architecture and EEG power spectra, and provide insight into the mechanisms underlying sleep abnormalities in individuals with AKI or CKD. Regarding sleep management in individuals with kidney failure, it is thus crucial to be aware of the possibility that kidney impairment directly causes sleep disturbances independent of treatment, comorbidities, or patient background.

## Introduction

Mammalian sleep is characterized by the cyclic occurrence of rapid eye movement sleep (REMS) and non-REMS (NREMS). In humans, NREMS is divided into three stages (N1, N2, and N3); N3 is also called slow-wave sleep due to the prominent slow-wave activity (SWA) in the electroencephalogram (EEG). N3 is thought to contribute to maintaining health and brain functions by increasing growth hormone secretion, suppressing corticosterone secretion, promoting synaptic plasticity, etc., and N3 amount is typically decreased in patients with depression (Gronfier et al., 1996; Yasugaki et al., 2025). REMS is characterized by muscle atonia, desynchronized cortical activity, and hippocampal theta oscillations. In addition, during REMS, cerebral capillary blood flow is largely elevated, which might also contribute to maintaining brain functions (Tsai et al., 2021). REMS Abnormalities in sleep architecture accompany various diseases (Cai et al., 2021; Chen et al., 2024; Iranzo et al., 2024; Yasugaki et al., 2025). Sleep disorders are common not only in individuals with mental or neurologic disorders but also in individuals with respiratory diseases, diabetes, cardiovascular disease, and kidney disease (Laverty et al., 2023; Madsen et al., 2019; McNicholas et al., 2019; Tan et al., 2022), implying that peripheral tissues can profoundly affect sleep.

Kidney impairment can be roughly classified into acute kidney injury (AKI) and chronic kidney disease (CKD). Both AKI and CKD are clinical syndromes characterized by changes in kidney function or structure and are associated with high morbidity, high mortality, and huge financial burden (Bikbov et al., 2020; Li and Li, 2021). AKI is defined as a rapid decline in kidney function, typically manifested by reduced urine output and electrolyte disturbances, and may progress to uremia. It is frequently observed in hospitalized patients, with particularly high incidence in intensive care units. CKD, on the other hand, is characterized by a persistent reduction in kidney function or structural abnormalities lasting more than three months, and is associated with complications such as anemia and increased cardiovascular risk. In advanced stages, CKD may progress to end-stage renal disease, necessitating dialysis or kidney transplantation (Stevens et al., 2024). Although AKI is generally considered a reversible form of kidney failure, recent studies found that AKI may develop into irreversible kidney fibrosis, which can trigger serious CKD (Niculae et al., 2023; Wang and Zhang, 2022), and the close relationship between AKI and CKD as part of a disease continuum is becoming increasingly recognized (Stevens et al., 2024). While data on the mechanisms driving kidney fibrosis are accumulating from animal model studies, effective therapeutic strategies to prevent or delay the transition from AKI to CKD have not yet been established (Niculae et al., 2023; Wang and Zhang, 2022). In this regard, the AKI-to-CKD transition is an important research focus in kidney disease.

In CKD, sleep disorders such as insomnia, obstructive sleep apnea syndrome, and restless legs syndrome are very commonly observed (Lyons, 2024; Tan et al., 2022). Whether sleep abnormalities are a direct consequence of kidney impairment, however, remains poorly understood. Sleep in individuals with CKD is affected by various factors, such as lifestyle-related diseases, obstructive sleep apnea syndrome, comorbidities such as depression, side effects of dialysis and symptomatic drugs, and poor sleep hygiene (ex. falling asleep during dialysis). Animal models allow us to study the impact of kidney impairment on sleep architecture, independent of the effectiveness of treatment of comorbidities and dialysis.

Aristolochic acid (AA) nephropathy is considered a drug-associated kidney injury disease (Michl et al., 2014). AA is transported into proximal tubule cells via basolateral organic anion transporters (OAT)1 and OAT3, where it induces the formation of DNA adducts. This triggers a p53-mediated DNA damage response, resulting in tubular injury that can progress to CKD, characterized by progressive interstitial fibrosis and loss of nephron function (Baudoux et al., 2022; Dickman et al., 2011; Michl et al., 2014; Navarro Garrido et al., 2022). Mice treated with AA are widely used as an animal model for studying the AKI-to-CKD transition. A single intraperitoneal injection of AA at a dose of 10 mg/kg in mice increases serum creatinine levels and causes severe kidney tubular damage within 3 days. Creatinine levels peak at day 14, and more obvious kidney tubular atrophy along with interstitial fibrosis are observed at day 28 (Kuang et al., 2021). Rats given AA orally exhibit AA-DNA adducts in the kidney and stomach but not in the brain (Schmeiser et al., 1988), suggesting that AA does not migrate directly to the central nervous system. Thus, the AA-induced AKI-to-CKD transition model is suitable for investigating the effects of kidney impairment on sleep. In the present study, we performed EEG recordings in mice undergoing AKI-to-CKD transition following a single intraperitoneal administration of AA and assessed the changes in sleep architecture caused by kidney impairment.

## Results

### Aristolochic acid induces kidney impairment in a dose-dependent manner in mice

To assess whether changes in the sleep architecture are associated with the AKI-to-CKD transition, we utilized the AKI-to-CKD mouse model induced by a single intraperitoneal dose of 5 or 10 mg/kg AA (Hu et al., 2021; Kuang et al., 2021; Wang et al., 2020). For control mice, only vehicle was administered. Following administration of the drug or vehicle into mice at the age of 10–11 weeks, sleep was monitored by 24-h EEG and electromyogram (EMG) recordings on days 6–7, representing the AKI stage, and days 27–28, representing the CKD stage. To confirm that kidney impairment was induced by AA administration, blood was drawn from the mice immediately after each sleep measurement to measure the serum creatinine levels. Experiments were performed in two independent cohorts of mice, each comprising vehicle, 5 mg/kg AA, and 10 mg/kg AA treatment groups, and the results between cohorts were combined. Mouse survival rates were 100% during the course of the study.

**Figure 1:**
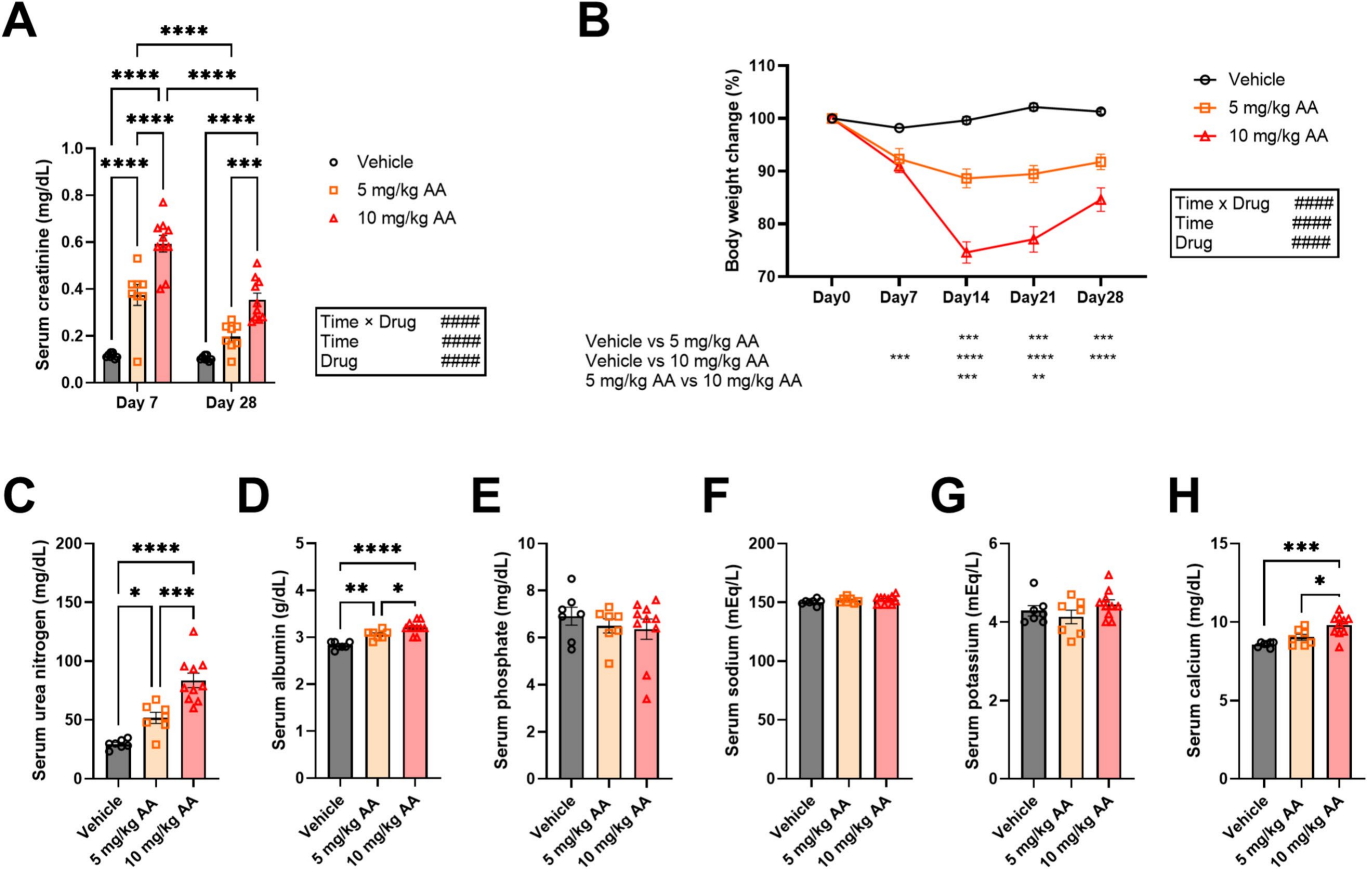
Aristolochic acid dose-dependently induces changes in serum creatinine and body weight, resembling the AKI-to-CKD transition. **(A)** Comparison of serum creatinine levels on day 7 (AKI) and day 28 (CKD). Each point represents an individual mouse. **(B)** Changes in body weight during the experiment. Body weight on the day of injection of aristolochic acid (day 0) was set as 100%. Each point represents the mean ± SEM. **(C–H)** Changes in serum levels of urea nitrogen **(C)**, albumin **(D)**, phosphate **(E)**, sodium **(F)**, potassium **(G)**, and calcium **(H)** on day 28 (CKD). Each point represents an individual mouse. Bar graphs represent mean ± SEM. (# indicates significance in two-way ANOVA. #*p* < 0.05; ##*p* < 0.01; ###*p* < 0.001; ####*p* < 0.0001. * indicates significance in Bonferroni’s test. *p < 0.05; **p < 0.01; ***p < 0.001; ****p < 0.0001).

Serum creatinine levels increased in an AA dose-dependent manner (Figure 1A). For both the 5 mg/kg and 10 mg/kg AA groups, the serum creatinine levels were lower on day 28 (CKD) compared with day 7 (AKI) (Figure 1A). Body weight also decreased in an AA dose-dependent manner. The body weight decrease peaked on day 14 and then exhibited a trend toward recovery (Figure 1B). These observations indicate that the well-established AKI-to-CKD mouse model with a single dose of 10 mg/kg AA was replicated in our study and that a single dose of 5 mg/kg AA caused milder symptoms.

Regarding the CKD mice, we also measured serum levels of additional biomarkers, i.e., urea nitrogen, albumin, phosphate, and electrolytes (e.g., sodium, potassium, and calcium). Consistent with a previous study (Hu et al., 2021), urea nitrogen was largely increased in an AA-dose-dependent manner (Figure 1C). We also observed increases in albumin and calcium, whereas phosphate, sodium, and potassium were not significantly affected (Figures 1D–H).

### AKI mice exhibit an increase in the total amount of NREMS

**Figure 2:**
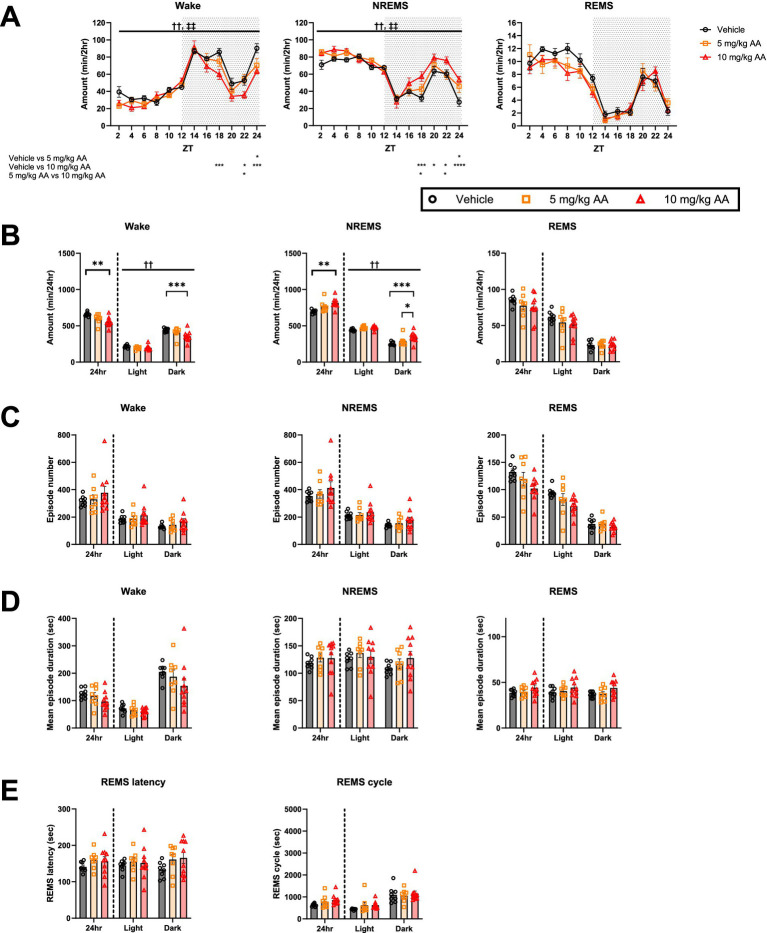
AKI mice exhibited an increase in the amount of NREMS. **(A)** Comparison of the daily variations in the amount of wake, NREMS, and REMS every 2 h on days 6–7 (AKI) between the vehicle, 5 mg/kg AA, and 10 mg/kg AAgroups. ZT zeitgeber time. Each point represents the mean ± SEM. **(B)** Comparison of the time spent in wake, NREMS, and REMS. **(C–E)** Comparison of the mean episode numbers **(C)** and durations **(D)** of each vigilance state, REMS latency, and REMS cycle **(E)**. Each point represents an individual mouse. († and ‡ indicate significant main effect of drug and significant interaction between drug and time, respectively, in two-way ANOVA. ††, ‡‡ *p* < 0.01. * indicates significance in Bonferroni’s test. *p < 0.05; **p < 0.01; ***p < 0.001; ****p < 0.0001).

Comparison of the sleep architecture in the 10 mg/kg AA, 5 mg/kg AA, and vehicle groups on days 6–7 (AKI) (Figure 2) revealed an increase in the total amount of NREMS and a concomitant decrease in the total amount of wake in the 10 mg/kg AA group (Figure 2B). These changes were attributed to changes in the dark (active) phase, especially the second half (Figures 2A,B). Although the difference between the 5 mg/kg AA group and the vehicle group was not statistically significant, a trend was observed, suggesting a dose-dependent effect of AA on the total amounts of NREMS and wake (Figure 2B). On the other hand, the total REMS amount did not differ significantly between the groups. In addition, neither the mean episode numbers and durations of each vigilance state nor the REMS latency and REMS cycles differed significantly between the groups (Figures 2A–E).

### CKD mice exhibit a normal amount of NREMS but a decrease in the amount of REMS

**Figure 3:**
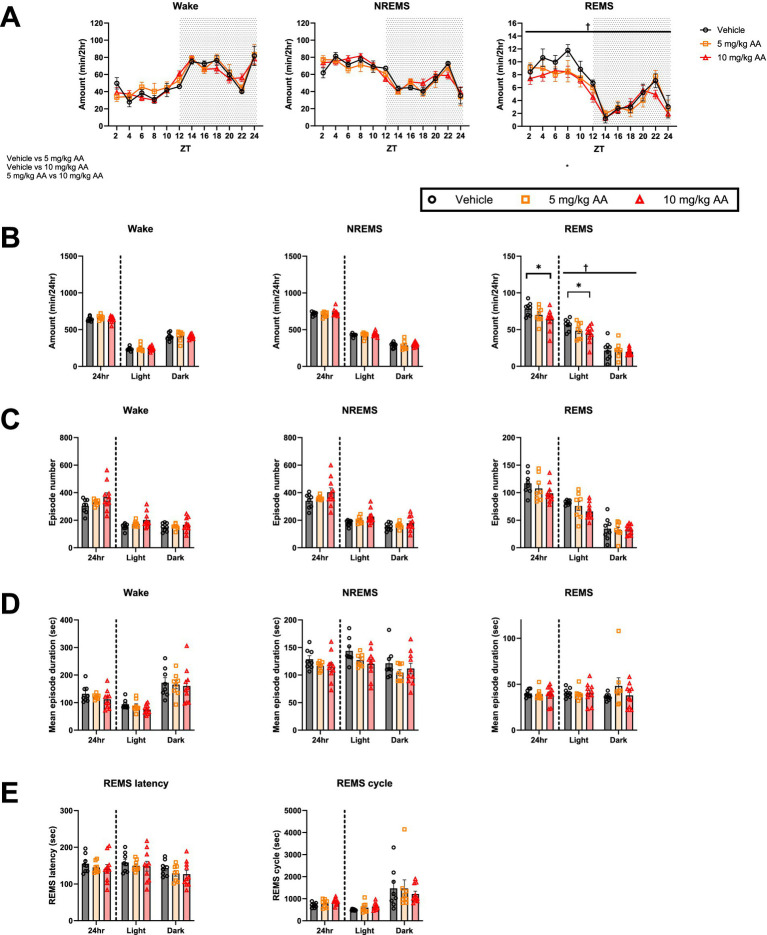
CKD mice exhibited a decrease in the amount of REMS. **(A)** Comparison of the daily variations in the amount of wake, NREMS, and REMS every 2 h on days 27–28 (CKD) between the vehicle, 5 mg/kg AA, and 10 mg/kg AA groups. ZT zeitgeber time. Each point represents the mean ± SEM. **(B)** Comparison of the time spent in wake, NREMS, and REMS. **(C–E)** Comparison of the mean episode numbers **(C)** and durations **(D)** of each vigilance state, REMS latency, and REMS cycle **(E)**. Each point represents an individual mouse. († and ‡ indicate a significant main effect of the drug and significant interaction between drug and time, respectively, in two-way ANOVA. †*p* < 0.05. * indicates significance in Bonferroni’s test. *p < 0.05).

In contrast to days 6–7 (AKI) (Figure 2), on days 27–28 (CKD) (Figure 3), we observed no significant difference in the total amounts of NREMS or wake between the groups (Figures 3A,B). In the 10 mg/kg AA group, however, the total amount of REMS, which was unaffected on day 6–7 (AKI), was reduced (Figure 3B). This was attributed to changes in the light (inactive) phase (Figures 3A,B). No significant differences were detected in the mean episode numbers and durations of each vigilance state or in the REMS latency and REMS cycles (Figures 3C–E).

### EEG delta power during NREMS is decreased in AKI, whereas theta power during wake and REMS is decreased in CKD

**Figure 4:**
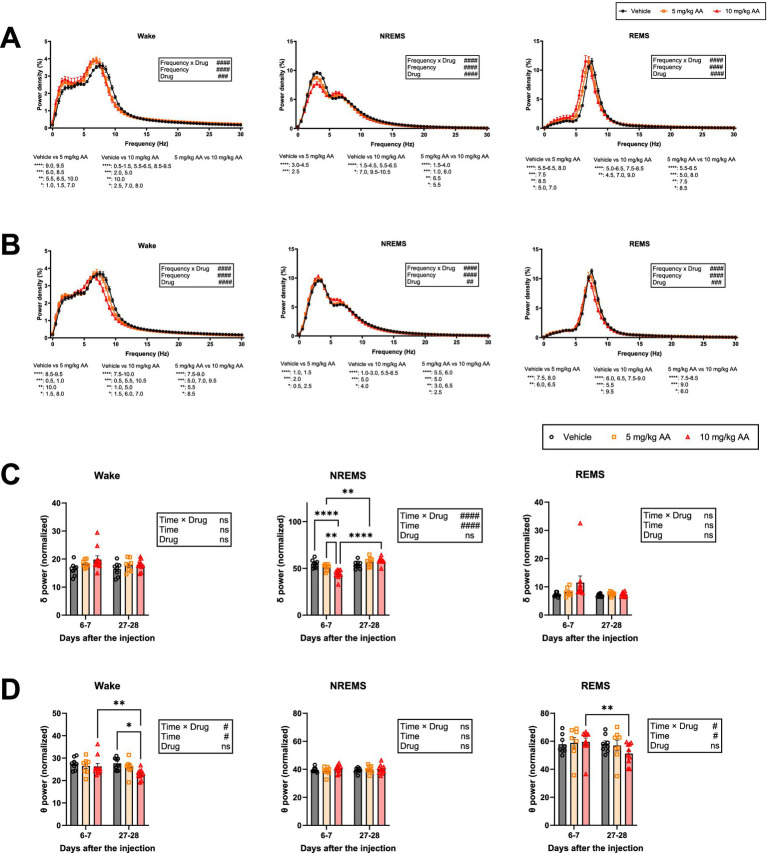
Mice exhibited a decrease in delta power during NREMS in AKI and in theta power during wake and REMS in CKD, and EEG slowing during wake and REMS throughout the AKI-to-CKD transition. **(A,B)** Comparison of the EEG power spectra of each vigilance state on days 6–7 (AKI) **(A)** and days 27–28 (CKD) **(B)**. Each point represents the mean ± SEM. C, D Comparison of delta (0.5–4 Hz) power **(C)** and theta (6-10 Hz) power **(D)** during each vigilance state on days 6–7 (AKI) and days 27–28 (CKD). Each point represents an individual mouse. (# indicates significance in two-way ANOVA. #*p* < 0.05; ##*p* < 0.01; ###*p* < 0.001; ####*p* < 0.0001. * indicates significance in Bonferroni’s test. *p < 0.05; **p < 0.01; ***p < 0.001; ****p < 0.0001).

We also examined the EEG power spectra during each vigilance state (Figure 4). On days 6–7 (AKI), the NREMS delta power was lower in the 10 mg/kg AA group compared with the vehicle group (Figure 4C), and the effect on the delta power seemed to be AA dose-dependent (Figure 4A). Decreased NREMS delta power reflects poorer sleep quality (Borbély and Achermann, 1999; Long et al., 2021). Thus, during AKI, although the amount of NREMS was increased, the quality was diminished.

In contrast to days 6–7 (AKI), on days 27–28 (CKD), the NREMS delta power in the AA-injected groups was not significantly different from that in the vehicle group (Figure 4C). The theta power during REMS and wake, however, was reduced in an AA dose-dependent manner (Figure 4A). On days 27–28 (CKD), the theta power during REMS and wake was decreased in the 10 mg/kg AA group compared to that on days 6–7 (AKI) (Figure 4D).

In addition, during both AKI and CKD, the peaks of the EEG power spectra during wake and REMS were shifted to a lower frequency (Figure 4A), a phenomenon known as EEG slowing.

### Sleep disturbances in AKI and CKD mice correlate with serum creatinine levels

**Figure 5:**
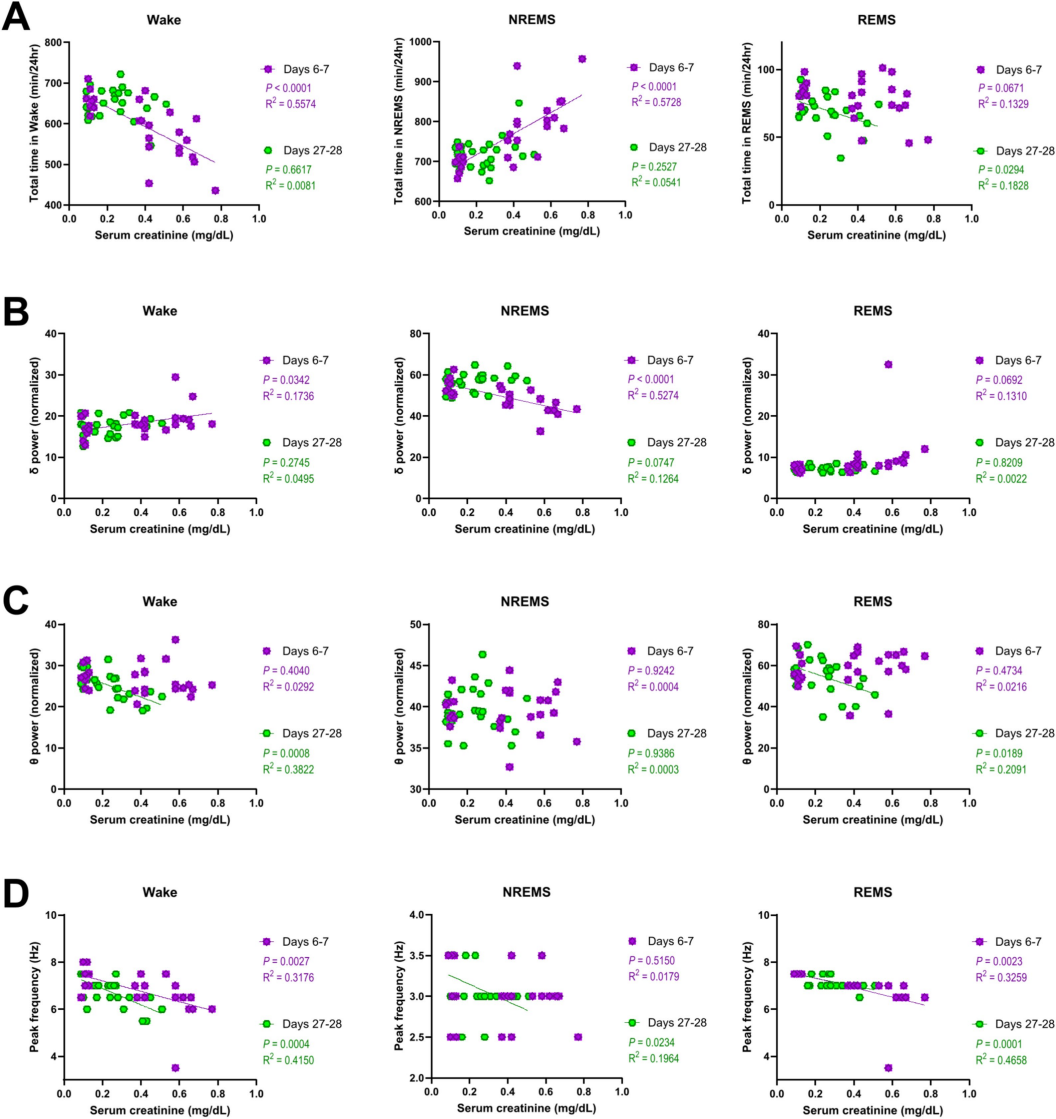
Sleep disturbances in AKI and CKD mice correlated with serum creatinine levels. Correlations between the serum creatinine levels and total amount of time **(A)**, EEG delta power **(B)**, theta power **(C)**, and peak frequency **(D)** of each vigilance state. Each point represents an individual mouse. The line in each graph represents the regression line. Pearson’s correlation coefficient (R^2^) and the *p* values are provided.

To investigate whether sleep abnormalities are associated with the degree of kidney impairment, we performed a correlation analysis between various sleep parameters and serum creatinine levels (Figure 5). On days 6–7 (AKI), both the decrease in the amount of wake and the increase in the amount of NREMS correlated with the serum creatinine levels (Figure 5A). The delta power of NREMS on days 6–7 (AKI) also negatively correlated with the serum creatinine levels (Figure 5B). On days 27–28 (CKD), the decrease in the amount of REMS correlated with the serum creatinine levels (Figure 5A), and the theta power of both wake and REMS negatively correlated with the serum creatinine levels (Figure 5C). Furthermore, on both days 6–7 (AKI) and days 27–28 (CKD), serum creatinine levels negatively correlated with the peak frequency of wake and REMS (Figure 5D).

### REMS disturbances in CKD mice also correlate with serum levels of urea nitrogen and calcium

**Figure 6:**
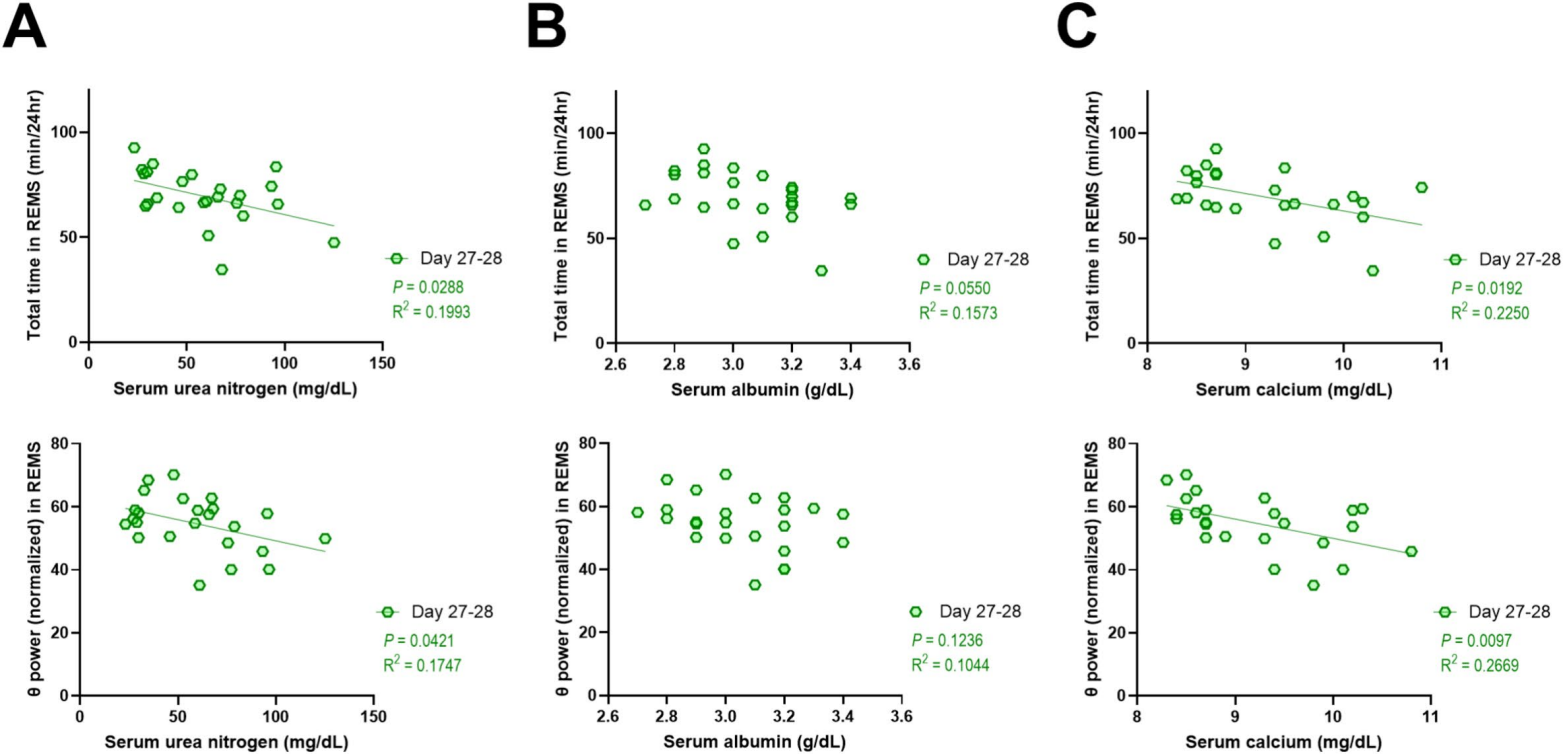
REMS disturbances in CKD mice also correlated with serum levels of urea nitrogen and calcium. Correlations between serum levels of urea nitrogen **(A)**, albumin **(B)**, or calcium **(C)** and total amount of REMS (top) and theta power during REMS (bottom). Each point represents an individual mouse. The line in each graph represents the regression line. Pearson’s correlation coefficient (R^2^) and the *p* values are provided.

Regarding the CKD mice, considering that there were increases in serum levels of additional biomarkers, i.e., urea nitrogen, albumin, and calcium, and considering that REMS was significantly affected, we additionally conducted correlation analyses between these biomarkers and the parameters of REMS. As a result, serum levels of urea nitrogen and calcium were negatively correlated with both the amount of REMS and the theta power during REMS (Figures 6A,C), whereas no significant correlations were detected for serum albumin levels (Figure 6B). In addition, no significant correlations were detected for serum levels of sodium, potassium, or phosphate (data not shown).

**Figure 7:**
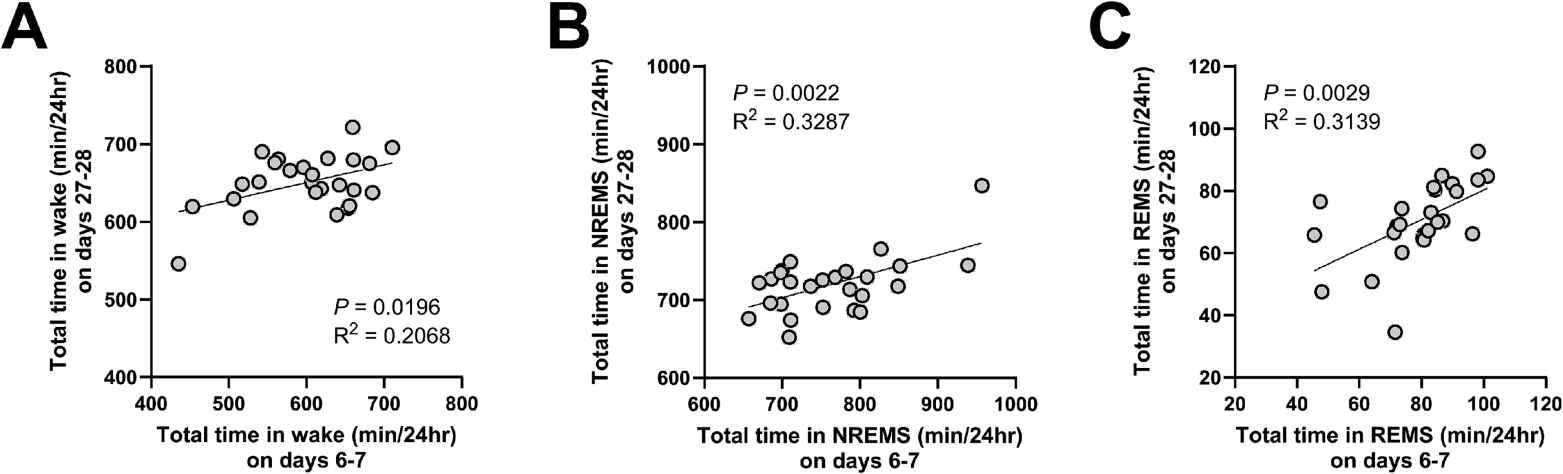
The amount of time in each vigilance state was positively correlated between AKI and CKD. Correlations in the total amount of wake **(A)**, NREMS **(B)**, and REMS **(C)**, between days 6–7 (AKI) and days 27–28 (CKD). Each point represents an individual mouse. The line in each graph represents the regression line. Pearson’s correlation coefficient (R^2^) and the *p* values are provided.

Finally, positive correlations were observed between the amount of each vigilance state on days 6–7 (AKI) and on days 27–28 (CKD) (Figure 7).

## Discussion

This rodent study is, to our knowledge, the first to examine the effect of kidney impairment on sleep architecture during the acute- to-chronic phase transition. While individuals with CKD often experience a high prevalence of poor sleep quality and insomnia (Tan et al., 2022), definitive evidence regarding the relationship between sleep disturbances and CKD has been limited due to the confounding effects of comorbidities and treatments. Using a mouse model free from such confounders, this study demonstrated that kidney dysfunction induces biphasic changes in sleep architecture and EEG power spectra in mice. In the acute phase (AKI), the amount of NREMS was increased, whereas EEG delta power, i.e., SWA in NREMS, was reduced. In the chronic phase (CKD), the NREMS abnormalities observed in the acute phase (AKI) recovered, whereas the amount of REMS was reduced, with EEG theta power showing a decrease during both wake and REMS. During both AKI and CKD, EEG slowing during wake and REMS was observed. These sleep abnormalities correlated with the serum creatinine levels, further supporting a strong association between kidney impairment and sleep disruption.

Sleep disturbances in individuals with CKD frequently co-occur with cardiovascular disease, mental disorders, and social and physical dysfunctions. The sleep changes observed in this study, however, were distinct from those reported in mouse models of cardiovascular disease or stress (see discussion below). Thus, kidney impairments may have direct effects on brain functions related to sleep and brain oscillation.

The sleep patterns of rats with 5/6 nephrectomy as a model of CKD were investigated previously (Hirotsu et al., 2010). Interpretation of the study results was limited, however, by the lack of a sham surgery control. In addition, surgical models of kidney dysfunction generally have surgical comorbidities and increased mortality. Therefore, changes in the sleep architecture during the AKI-to-CKD transition have remained unclear. Although previous studies using adenine-induced nephropathy mouse models did not measure EEG or examine detailed sleep architecture, they reported fragmented sleep and activity rhythms assessed by infrared sensor and video monitoring, which might not yield accurate sleep/wake judgments (Motohashi et al., 2020). Mice fed an adenine-containing diet for more than 2 weeks showed increased urination and drinking behaviors, behavioral changes that could lead to sleep fragmentation. In contrast, the AA-injected mice in our study exhibited no significant differences in the number or duration of sleep episodes between the vehicle and AA groups during either the acute or chronic phase, suggesting that the observed sleep changes were not merely consequences of behavioral alterations.

During the acute phase, AA-treated mice exhibited increased total NREMS time but the SWA during NREMS was reduced, i.e., AKI mice have a greater amount of NREMS, but the quality of the NREMS is poorer. Mouse models of systemic inflammation induced by the administration of lipopolysaccharides (LPS) also exhibit an increased amount of NREMS but reduced EEG SWA (Valencia-Sanchez et al., 2024). Thus, the changes in NREMS in AKI mice might be due to systemic inflammation. Indeed, AA-treated mice exhibit increased levels of pro-inflammatory mediators such as interleukin-1β during the acute phase (Hu et al., 2021). Considering that benzodiazepines also increase NREMS while suppressing EEG SWA (McKillop et al., 2021), NREMS alterations caused by AKI (or systemic inflammation in general) might result from a global change in the excitation-inhibition balance.

A remarkable feature of our AKI-to-CKD mouse model was the large decrease in the REMS amount during the chronic phase (CKD), which is difficult to explain with existing models of REMS regulation. Reduced REMS, together with the decreased theta power during both REMS and wake, may result from uremic toxin accumulation due to kidney dysfunction. Recent studies identified neurons in the brainstem (including the pons and medulla) that regulate REMS, including neurons that strongly promote or inhibit REM sleep (Hayashi et al., 2015; Kashiwagi et al., 2024). Some of these neurons are not required under basal conditions but are involved in alterations in REM sleep under stressful conditions (Liu et al., 2021). In individuals with CKD not undergoing dialysis, uremic toxins accumulate in various brain regions, including the brainstem (De Deyn et al., 1995). Uremic toxins may adversely affect these neurons, either causing hypoactivity of REMS-promoting neurons or hyperactivity of REMS-inhibiting neurons and thus leading to decreased REMS. Theta rhythms, generated in the hippocampus, dominate hippocampal activity during active wakefulness and REMS in rodents (Winson, 1974). Theta rhythms are affected by various subcortical areas, including the brainstem, and theta rhythm attenuation might also be due to uremic toxin accumulation. In our study, REM sleep reduction and attenuation of theta oscillations were correlated with serum levels of creatinine, urea nitrogen, and calcium, whereas no correlations were observed for albumin, phosphate, sodium, or potassium. These results may provide hints to unveil the responsible substances.

Notably, changes in sleep associated with the AKI-to-CKD transition cannot be explained by stress. In a study evaluating the effect of chronic stress on sleep in mice, exposure to water immersion and restraint stress led to a large decrease in wake and an increase in both NREMS and REMS in the first week that was less pronounced in the second or third week despite continued exposure to stress (Yasugaki et al., 2019). Chronic exposure to glucocorticoids, which increase during stress, also increases the REMS amount (Yao et al., 2023). The sleep phenotypes of our AKI-to-CKD mouse model are also distinct from phenotypes of mouse models of cardiovascular disease, in that cardiovascular disease models show both increased NREMS amount and EEG SWA (Huynh et al., 2024), further indicating that a unique mechanism underlies the sleep alterations associated with kidney impairment.

Another noteworthy feature of the AA-injected mice was the EEG slowing observed during REMS and wake throughout the AKI-to-CKD transition. Similar symptoms are observed in individuals with uremic encephalopathy (Bourne et al., 1975). EEG abnormalities are also observed in individuals with CKD without overt clinical signs of uremic encephalopathy (Gadewar et al., 2015). In individuals with non-dialysis kidney failure, the degree of EEG slowing during wake correlates with the creatinine levels (Bourne et al., 1975), consistent with a direct effect of kidney dysfunction on brain functions related to brain activity oscillations. EEG abnormalities may serve as indicators of the effects of kidney dysfunction on the brain. EEG slowing and reduced REM sleep amount are also observed in individuals with Alzheimer’s disease or Alzheimer’s disease mouse models (Maezono et al., 2020; Petit et al., 1993). REMS is associated with a marked increase in cerebral capillary blood flow, and its reduction may impair substance exchange via the capillaries, potentially contributing to brain dysfunction (Tsai et al., 2021). Interestingly, numerous studies have linked kidney dysfunction to cognitive impairment. Individuals with CKD frequently exhibit cognitive dysfunction (Hamed, 2019), with epidemiologic studies associating reduced kidney function levels in individuals having CKD with cognitive decline (Khatri et al., 2009; Kurella et al., 2005; Yaffe et al., 2010). Individuals with AKI are also at significantly increased risk of developing dementia, suggesting potential long-term adverse effects (Tsai et al., 2017). Previous studies reported hippocampal neurodegeneration and memory deficits in other kidney dysfunction animal models, such as adenine-treated rats (Watanabe et al., 2021) and 5/6 nephrectomy mice (Fujisaki et al., 2014). The reduction in REMS and theta power may be involved in mechanisms linking kidney dysfunction and brain impairment.

In conclusion, this study revealed that AA-induced kidney impairment in a mouse model of AKI-to-CKD leads to biphasic sleep changes comprising acute and delayed phases. These findings demonstrate the impact of kidney dysfunction on sleep/wake regulation, independent of comorbidities and treatments such as dialysis that were previously inseparable from CKD. Furthermore, the sleep changes observed in this study differ from those reported in models of stress, infection, inflammation, and cardiovascular disease, offering novel insights into disease-associated sleep disturbances. These findings suggest the existence of kidney function-related pathways regulating sleep architecture.

## Limitations of the study

This study has several limitations. First, serum levels of only a few biomarkers were measured. Previous studies on AA-induced AKI-to-CKD mice conducted comprehensive analyses, including other kidney function indicators (e.g., urinary protein), assessments of structural kidney damage, tubular epithelial cell atrophy, collagen fiber deposition in the tubulointerstitial area, fibrosis markers, and inflammatory factors (Hu et al., 2021; Kuang et al., 2021; Wang et al., 2020). These studies confirmed AKI and CKD findings on days 7 and 28, respectively, following a single administration of 10 mg/kg AA, validating serum creatinine as a key indicator. Further research is needed to explore the relationship between AKI or CKD pathophysiology and sleep changes. Second, only male mice were used in this study, as female mice are less susceptible to AA-induced kidney injury (Huang et al., 2013; Li et al., 2023). Additional studies investigating potential sex differences in sleep changes related to kidney injury are necessary. Third, the mechanisms linking kidney injury to sleep changes remain unclear. We solely used the AA-induced AKI-to-CKD transition model, which represents a specific pathophysiological mechanism and may not recapitulate the broader spectrum of kidney diseases. In addition, assessment of the correlational analyses was limited to a few biomarkers. Additional studies on how sleep deficits correlate with other dysfunctions resulting from kidney failure such as hormonal imbalance, cognitive impairment, and autonomic dysfunctions may help improve our understanding of sleep disturbances associated with kidney injury.

## Methods

### Animals and drug treatment

All animal experiments were approved by the institutional animal care and use committee of the Kyoto University Graduate School of Medicine. Male C57BL/6JJcl mice (8 weeks of age) were purchased from Clea Japan (Tokyo, Japan). The mice were individually housed under a 12/12 h light/dark cycle (lights on at 8.30 am) at a controlled temperature (21.0 ± 2.0°C) with free access to water and food.

The mice were divided into the following groups, avoiding weight bias between groups: vehicle group (*n* = 8), 5 mg/kg AA group (*n* = 8), and 10 mg/kg AA group (*n* = 10). The AA groups were intraperitoneally injected with AA-I sodium salt (A9451, Sigma-Aldrich, St. Louis, MO, USA) which was dissolved in a small amount of saline solution (9K90S, Otsuka Pharmaceutical Factory, Inc., Japan). The vehicle group was intraperitoneally injected with an equal volume per body weight of saline solution. Drug treatment was administered as a single dose when the mice were 10 to 11 weeks of age.

### Sleep recording

Male mice were monitored by EEG/EMG recording to characterize the sleep architecture. Under isoflurane anesthesia, 8-week-old mice were implanted with EEG/EMG electrodes. Stainless steel EEG electrodes were implanted epidurally over the parietal cortex (3 mm posterior to bregma, 1.5 mm lateral to the midline) and cerebellum (6.5 mm posterior to bregma, 2 mm lateral to the midline), and EMG electrodes were embedded into the trapezius muscles bilaterally. Mice were allowed to recover from surgery in their home cage for at least 14 days. When mice were 11 to 12 weeks of age (AKI) or 14 to 15 weeks of age (CKD), they were placed in a sleep recording chamber. After habituation for at least 6 days, EEG and EMG signals were recorded from freely moving mice in accordance with the previously described method (Hayashi et al., 2015).

EEG/EMG signals were recorded from the onset of the light phase for 24 h. EEG/EMG signals were band-pass filtered at 0.5–128 Hz, collected, and digitized at a sampling rate of 256 Hz using VitalRecorder (Kissei Comtec, Nagano, Japan). The EEG signals were divided into 4-s epochs, subjected to fast Fourier transform, and further analyzed using SleepSign (Kissei Comtec). The vigilance state in each epoch was manually classified as REMS, NREMS, or wake based on the EEG patterns, the absolute delta (0.5–4 Hz) power, the theta (6–10 Hz) power to delta power ratio, and the integral of the EMG signals. Epochs with high EMG and low delta power were classified as wake, epochs with high delta power and low EMG were classified as NREMS, and epochs with even lower EMG (suggestive of muscle atonia) and a high theta power to delta power ratio were classified as REMS. If a single epoch contained multiple states, the predominant state was assigned. Epochs that contained presumable large movement-derived artifacts in the EEG data were included in the stage analysis but were excluded from the EEG power spectrum analysis. When calculating the EEG power spectrum of wake, NREMS, or REMS, the EEG power of each frequency bin was expressed as a percentage of the mean total EEG power over all frequency bins (1–30 Hz) across 24 h. All scoring was performed by an experimenter blinded to the experimental groups. REMS latency was defined as the average duration of the NREMS episode immediately prior to each REMS episode, and the REMS cycle was defined as the average time from the onset of a REMS epoch to the onset of the next REMS epoch.

### Blood samples

Blood samples were collected after recording sleep on experiment day 7 and day 28. On day 7, blood samples were collected through the retroorbital venous sinus using a glass capillary (Microcaps 30 μL, 1–000-0300, Drummond Scientific Company, Broomall, PA, USA) under isoflurane anesthesia. On day 28, blood samples were collected by cardiac puncture under sevoflurane anesthesia and mice were subsequently killed by an overdose of anesthesia. After blood collection, the blood was mixed thoroughly by gentle inversion 5–6 times and placed at room temperature for approximately 80 min. Whole blood samples were centrifuged at 1200G (MDX-310; Tomy Seiko Co., Ltd., Tokyo, Japan) for 10 min at room temperature to separate the serum. The resultant serum samples were stored at −30°C. Serum levels of creatinine, urea nitrogen, albumin, phosphate, sodium, potassium, and calcium were measured using a Hitachi 7,180 autoanalyzer (Hitachi, Ltd., Tokyo, Japan).

### Statistical analyses

Statistical analyses were performed using PRISM 8 (GraphPad Software, Boston, MA, USA), and statistical significance was set at *p* = 0.05. Bar graphs and line graphs represent mean ± standard error of the mean (SEM). Each point on the bar graphs and scatter plots represents an individual mouse. Comparisons between each group of mice or between AKI and CKD were conducted using Bonferroni’s test or two-way ANOVA followed by Bonferroni’s test. For the correlation analysis, Pearson’s correlation test was performed. Where applicable, all tests were two-tailed. For correlation analyses, Pearson’s correlation coefficient (R^2^) and the *p* values were calculated.

## Significance statement

Individuals with kidney failure have higher dementia risk, underscoring the importance of understanding how kidney impairment affects the brain. While sleep disorders are common in individuals with kidney failure, sleep abnormalities that emerge as direct consequence of kidney impairment have remained unknown. By utilizing a mouse model of acute kidney injury-to-chronic kidney disease transition, we demonstrated that kidney failure has distinct effects on sleep during acute and chronic phases. In particular, REM sleep, which is the major source of vivid dreams, is largely reduced during the chronic phase. Reduced REM sleep is a risk factor for dementia and mortality, indicative of its critical role in health. Our results present a novel peripheral mechanism by which REM sleep is significantly impaired.

## Data Availability

The datasets presented in this study can be found in online repositories. The names of the repository/repositories and accession number(s) can be found at: https://doi.org/10.15083/0002010562.
